# Clinical Evaluation of the Clinical Reasoning Process of Large Language Models in Nephrology: Comparative Evaluation Study

**DOI:** 10.2196/89726

**Published:** 2026-06-03

**Authors:** Yuichiro Yano, Hiroaki Kakizaki, Hajime Nagasu, Seiji Kishi, Takeo Koshida, Yoshihito Nihei, Akira Hirano, Masaomi Nangaku, Hirotake Mori, Toshio Naito, Mizuki Ohashi, Shoichi Maruyama, Isao Matsui, Yoshitaka Isaka, Hirokazu Okada, Yusuke Suzuki, Naoki Kashihara

**Affiliations:** 1 Department of General Medicine, Juntendo University, 2-1-1, Hongo, Bunkyo-Ku, Tokyo, 113-8421, Japan, 81 3-3813-3111; 2 Artificial Intelligence Incubation Farm, Faculty of Medicine, Juntendo University, Tyoko, Japan; 3 PeopleMedia, Inc, Osaka, Japan; 4 Department of Nephrology and Hypertension, Kawasaki Medical School, Kurashiki, Okayama, Japan; 5 Department of Nephrology, Juntendo University, Tokyo, Japan; 6 Division of Nephrology and Endocrinology, The University of Tokyo, Tokyo, Japan; 7 Department of Nephrology, Nagoya University, Nagoya, Aichi, Japan; 8 Department of Nephrology, The University of Osaka, Suita, Osaka, Japan; 9 Department of Nephrology, Saitama Medical University, Saitama, Japan

**Keywords:** artificial intelligence, large language models, clinical reasoning, nephrology, evaluation

## Abstract

This study evaluates the dynamic clinical reasoning of 4 leading large language models in complex nephrology cases, demonstrating that while Gemini 2.5 Pro achieved the highest reasoning scores and computational efficiency, all tested models excelled at static data synthesis but shared vulnerabilities in formulating nuanced differential diagnoses and in prospective clinical planning.

## Introduction

While large language models (LLMs) are increasingly being applied in medicine, evaluating their performance relies on static knowledge tests, such as medical licensing examinations, which fail to capture the dynamic, iterative reasoning of real clinical practice [1,2]. Recent benchmark studies have begun to address this gap; for instance, the MedR-Bench [3] framework evaluates medical LLMs across 3 clinical stages using an automated, artificial intelligence (AI)–driven “reasoning evaluator” to score free-text reasoning. However, while automated metrics provide scalability, they cannot fully replace rigorous human verification in medical contexts.

The aim of this study is to perform a clinical evaluation of 4 leading LLMs (GPT-o3 [OpenAI], Gemini 2.5 Pro [Google], DeepSeek-R1 [Hangzhou DeepSeek Artificial Intelligence], and Llama 4 Maverick [Meta]) in nephrology [4], a specialty renowned for its complex, multisystemic pathologies and diagnostic challenges, using a multiagent architecture for temporal workflows [5]. Rather than using clinical reasoning with broad automated metrics, we systematically deconstructed the reasoning process into 9 distinct, scorable cognitive steps mapped to real-world workflows.

## Methods

### Overview

A detailed description of the methods is provided in Multimedia Appendix 1. Briefly, 4 nephrologists used the Delphi method to select 10 cases that met the inclusion criteria from over 100 case reports [6]. As permission could not be obtained for 1 case, 9 cases were included in the final analysis.

We developed a clinical reasoning application using Dify [7], a no-code AI development platform that enables the creation of AI agents leveraging LLMs via application programming interface (API) integration. We evaluated 4 LLMs: DeepSeek-R1 (DeepSeek-R1-0528, released May 28, 2025), Gemini 2.5 Pro (preview 03‐25, released May 25, 2025), GPT-o3 (released April 16, 2025), and Llama 4 Maverick (meta-llama/Llama-4-Maveric-17B-128E-Instruct-FP8, released July 20, 2025). All were accessed via the together.ai [8] API. All 4 LLMs used the same sequential 3-agent architecture designed to mirror the temporal progression of clinical practice. To systematically evaluate model performance, we deconstructed clinical reasoning into 9 cognitive steps (Table 1).

The evaluation of the LLM outputs was conducted on July 20, 2025. The primary outcome was the reasoning quality score, measured on a 3-point scale (0=incorrect; 1=reasonable but suboptimal; 2=correct). The 4 nephrologists independently and blindly scored the randomized, deidentified outputs. Results were aggregated by blinded researcher. Group differences were assessed using the Kruskal-Wallis test, followed by pairwise comparisons with Holm correction for multiple testing. A 2-sided *P* value <.05 was considered statistically significant. Interrater reliability was assessed using the intraclass correlation coefficient (ICC[2,k]). Full prompts and system details are available in Multimedia Appendix 1.

**Table 1.** End-to-end multi-agent clinical reasoning workflow and evaluated tasks. All agents operated under a strict system prompt with instructions to act as a “Japanese nephrologist specialized in clinical reasoning and evidence-based medicine,” thinking step-by-step in English while strictly relying only on provided information without assuming new facts.

Agent	Clinical stage	Input data	Nine cognitive steps and evaluated specific reasoning tasks (user prompts)	Expected output format and constraints (assistant prompts)
Agent 1	Initial clinical assessment	Patient’s chief concern and brief initial clinical findings	Step 1: medical summarization; question: “Summarize the patient as a medical problem.” Step 2: differential diagnosis; question: “Provide three differential diagnoses (most likely & ’must not miss’).” Step 3: additional data acquisition: question: “Suggest additional questions or physical exams to verify hypotheses.” Step 4: diagnostic planning; question: “Recommend diagnostic tests with purposes and expected outcomes.”	Include age, sex, and time course; present a prioritized differential list with rationale; list purposeful questions or examinations to rule in or out; develop a cost-effective and logical testing plan
Agent 2	Diagnostic refinement	Newly obtained diagnostic test results	Step 5: results interpretation; question: “Interpret test results in the context of the patient’s condition.” Step 6: diagnostic updating; question: “Update the diagnostic thinking and suggest the most likely working diagnosis.” Step 7: treatment formulation; question: “Propose a treatment plan with rationale and alternative options.”	Explain medical significance beyond mere abnormal values; appropriately revise the prioritization of differentials; develop a treatment plan considering both evidence-based medicine and patient factors
Agent 3	Therapeutic evaluation	Information on treatments administered and subsequent clinical outcomes	Step 8: monitoring and risk mitigation; question: “Propose strategies to monitor treatment effectiveness, specific risks, and countermeasures.” Step 9: contingency planning; question: ”Recommend actions if the condition fails to improve or changes suddenly.”	List specific measures for evaluating efficacy and side effects; explain the thought process for rapidly reassessing causes and flexibly revising the plan

### Ethical Considerations

Under the Ethical Guidelines for Medical and Biological Research Involving Human Subjects in Japan, this study was exempt from institutional review board review and informed consent requirements, as it exclusively involved the secondary analysis of fully anonymized, publicly available case reports without accessing personal health information.

## Results

**Figure 1.**
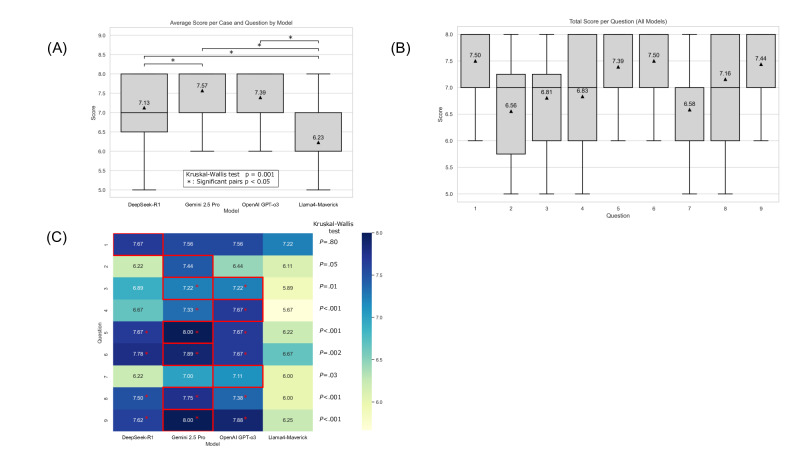
Scores by model for each case and question, and distribution of scores by clinical reasoning question. (A) Scores for each case and question are displayed by model as box-and-whisker plots. Overall differences were assessed with the Kruskal–Wallis test; when significant, pairwise comparisons between models were performed with Holm correction for multiple testing. Asterisks indicate a statistically significant difference (2-sided *P* value <.05) in performance compared to Llama 4 Maverick. (B) Box-and-whisker plots show the distribution of scores for each of the 9 clinical reasoning questions, aggregating the results from all 4 LLMs. The boxes represent IQRs, the lines inside the boxes indicate the medians, and the whiskers show the ranges of the data. Triangles mark the mean score for each question. The questions from 1 to 9 were as follows: summary of the medical problem; differential diagnoses and rationale; necessary physical examinations and rationale; plan for investigations/tests; interpretation of test results; reassessment of the differential diagnoses; treatment planning; evaluation of treatment; and management in case of clinical worsening. (C) The values presented as a heatmap, where red outlines highlight the highest-scoring model for each question. Group differences were assessed using the Kruskal-Wallis test, followed by pairwise comparisons with Holm correction for multiple testing.

Overall performance differed significantly among the 4 LLMs (Figure 1A). Gemini 2.5 Pro achieved the highest average score (mean 7.57, SD 0.61), followed by GPT-o3 (mean 7.39, SD 0.81), DeepSeek-R1 (mean 7.13, SD 1.03), and Llama 4 Maverick (mean 6.23, SD 0.93). Across all models (Figure 1B), Q2 and Q7 were the most challenging tasks, with the lowest mean scores (mean 6.56, SD 1.13 and mean 6.58, SD 0.97, respectively), while the models performed best on Q1 and Q6 (mean 7.50, SD 0.77, and mean 7.50, SD 0.85, respectively). Figure 1C shows a heatmap of average scores by model for each clinical reasoning question. Gemini 2.5 Pro demonstrated superior or competitive performance, particularly on complex tasks such as Q6 (mean 7.89, SD 0.85), Q5 (mean 8.00, SD 0.87), and Q9 (mean 8.00, SD 0.88). The ICC(2,k) was 0.36 (95% CI 0.24‐0.46). Gemini 2.5 Pro was efficient (mean response time: 124.7, SD 16.0 sec), whereas DeepSeek-R1 incurred the highest computational cost, with the longest response time (mean 249.7, SD 68.6 sec) and highest token use (mean 16,105.1, SD 4508.5) (Figure S1 in Multimedia Appendix 1). In contrast, Llama 4 Maverick had the shortest response time and the lowest token use.

## Discussion

We demonstrate that while LLMs effectively processed clinical data, overall performance varied significantly among models. Gemini 2.5 Pro achieved the highest overall reasoning quality score while maintaining computational efficiency. Our step-by-step evaluation revealed a consistent pattern across all models: while they excelled at information synthesis and test interpretation (questions 1 and 6), they shared specific vulnerabilities in higher-order cognitive tasks, particularly in formulating nuanced differential diagnoses (question 2) and planning optimal interventions (question 7).

Recent benchmarks, such as AMIE, MAI-DxO, and MedR-Bench [1-3,9], have advanced AI evaluation from static examinations toward dynamic clinical workflows. However, these frameworks often assess reasoning using broad, automated metrics. By deconstructing clinical reasoning into a stage-gated, multiagent workflow evaluated by domain experts, our study pinpoints exact cognitive bottlenecks. Our results indicate that LLMs struggle heavily with the higher-order, divergent skills required for prospective planning [10]. For example, when a nephrotic syndrome patient developed sudden-onset nocardiosis, the models failed to adequately pivot their proposed treatment plans. This exposes a critical gap between static knowledge retrieval and the fluid, iterative reality of complex multisystemic specialties like nephrology.

Furthermore, our findings challenge the assumption that bigger models are always better. We revealed a nuanced relationship between reasoning quality and computational efficiency. For real-world clinical deployment, where rapid bedside decision-making and sustainable API costs are paramount, identifying models that balance high reasoning quality with low computational overhead is an encouraging and practical metric.

A primary limitation is the small sample size of 9 nephrology cases evaluated across 9 specific questions, which restricts the generalizability of our findings across diverse, real-world clinical scenarios. However, the cases were rigorously curated via Delphi consensus from 104 candidates, prioritizing diagnostic complexity over breadth. By deconstructing these cases to generate 324 expert-scored data points per model, this granular approach might provide information power to detect statistically significant performance differences. Additionally, our reliance on expert-scored evaluation showed limited interrater agreement, and the study currently lacks extensive statistical validation. Our findings should be interpreted as an early, exploratory assessment of AI clinical reasoning rather than a definitively generalizable conclusion.

Ultimately, future model development in the medical field must move beyond static examinations to prioritize dynamic adaptability and prospective clinical planning. By emphasizing targeted cognitive evaluations and computational efficiency over sheer model size, the medical community can ensure the safe and sustainable integration of AI into real-world bedside practice.

## Supplementary material

10.2196/89726 Multimedia Appendix 1 Delphi case selection process, multiagent system architecture, prompt design, and comprehensive model responses across 9 structured clinical cases.

## Abbreviations

AI: artificial intelligence
API: application programming interface
LLM: large language model
